# Cognitive Function and Health Literacy Decline in a Cohort of Aging English Adults

**DOI:** 10.1007/s11606-015-3206-9

**Published:** 2015-02-14

**Authors:** Lindsay C. Kobayashi, Jane Wardle, Michael S. Wolf, Christian von Wagner

**Affiliations:** 1 Health Behaviour Research Centre, Department of Epidemiology and Public Health, University College London, 1-19 Torrington Place, 2nd floor, London, UK WC1E 6BT; 2 Division of General Internal Medicine, Feinberg School of Medicine, Northwestern University, Chicago, IL 60611 USA

**Keywords:** health literacy, cognition, aging, epidemiology

## Abstract

**BACKGROUND:**

Low health literacy is common among aging patients and is a risk factor for morbidity and mortality. We aimed to describe health literacy decline during aging and to investigate the roles of cognitive function and decline in determining health literacy decline.

**METHODS:**

Data were from 5,256 non-cognitively impaired adults aged ≥ 52 years in the English Longitudinal Study of Ageing. Health literacy was assessed using a four-item reading comprehension assessment of a fictitious medicine label, and cognitive function was assessed in a battery administered in-person at baseline (2004–2005) and at follow-up (2010–2011).

**RESULTS:**

Overall, 19.6 % (1,032/5,256) of participants declined in health literacy score over the follow-up. Among adults aged ≥ 80 years at baseline, this proportion was 38.2 % (102/267), compared to 14.8 % (78/526) among adults aged 52–54 years (OR = 3.21; 95 % CI: 2.26–4.57). Other sociodemographic predictors of health literacy decline were: male sex (OR = 1.20; 95 % CI: 1.04–1.38), non-white ethnicity (OR = 2.42; 95 % CI: 1.51–3.89), low educational attainment (OR = 1.58; 95 % CI: 1.29–1.95 for no qualifications vs. degree education), and low occupational class (OR = 1.67; 95 % CI: 1.39–2.01 for routine vs. managerial occupations). Higher baseline cognitive function scores protected against health literacy decline, while cognitive decline (yes vs. no) predicted decline in health literacy score (OR = 1.59; 95 % CI: 1.35–1.87 for memory decline and OR = 1.56; 95 % CI: 1.32–1.85 for executive function decline).

**CONCLUSIONS:**

Health literacy decline appeared to increase with age, and was associated with even subtle cognitive decline in older non-impaired adults. Striking social inequalities were evident, whereby men and those from minority and deprived backgrounds were particularly vulnerable to literacy decline. Health practitioners must be able to recognize limited health literacy to ensure that clinical demands match the literacy skills of diverse patients.

**Electronic supplementary material:**

The online version of this article (doi:10.1007/s11606-015-3206-9) contains supplementary material, which is available to authorized users.

## INTRODUCTION

In North America, over half of all adults and over 70 % of adults aged over 65 years have low health literacy, defined as having trouble accessing, understanding, and using information to make basic health decisions.^1^,^2^ Low health literacy is associated with taking of prescription medications improperly, excess use of emergency care, less use of preventive care services, and increased risks for morbidity and mortality.^3^–^7^ Evidently, there is a broad mismatch between individuals’ literacy skills and the health management demands placed upon them by health systems, resulting in literacy-based barriers to good health. The improvement of health literacy of populations is a major goal of health organizations including the U.S. Centers for Disease Control and Prevention and the World Health Organization.^8^,^9^

The health consequences of low literacy may be especially pertinent in older populations, given that older adults commonly need health information and services to manage their increasingly complicated health issues.^10^ Cross-sectional research has consistently associated older age with poorer performance on health literacy tests.^11^ Subsequently, health literacy skills are assumed to decline during aging. An important consideration for examining the dynamics of health literacy decline in older populations is cognitive aging, as cognitive function is related to health literacy. Fluid cognitive abilities (e.g., working memory, reasoning) and crystallized cognitive abilities (e.g., vocabulary, generalized knowledge) have been shown to jointly explain over 70 % of the association between health literacy and performance on health-related tasks among older adults.^12^

However, the effect of typical cognitive aging processes on health literacy skills remains unclear.^13^–^19^ Furthermore, the distribution of health literacy skill decline in an older population has never been demonstrated, an awareness of which would be imperative for researchers, health practitioners, and policymakers, because low health literacy is a major determinant of morbidity and mortality in the United States, England, and globally.^5^,^6^,^20^

In this study, we aimed to describe health literacy decline during aging and the potential contributing roles of cognitive function and decline to health literacy decline among non-cognitively impaired English adults aged ≥ 52 years.

## METHODS

### The English Longitudinal Study of Ageing

The English Longitudinal Study of Ageing (ELSA) is a population-based longitudinal cohort study that aims to characterize the economic, social, and health consequences of aging among English adults aged ≥ 50 years.^21^ The original ELSA cohort of 12,100 adults (response rate = 66 %) was established in 2002 based on a random stratified sample of households.^21^ ELSA data are collected biennially through computer-assisted interviews. The ELSA was approved by the London Multicentre Research Ethics Committee (MREC/01/2/91) and informed consent was obtained from all participants.

### Study Sample

All ELSA participants from the original cohort who were in the study at waves 2 (2004–2005) and 5 (2010–2011) of data collection were eligible for inclusion. Wave 2 included 8,780 participants of the original 12,100. Of these, 5,840 were retained at wave 5 (33.5 % attrition between waves 2 and 5) and were eligible for inclusion in the present analysis. Of the 8,780 participants at wave 2, 8,316 (94.7 %) completed the health literacy assessment. Common non-completion reasons were sight difficulties (n = 132), health problems (n = 59), or that the interview was done by proxy due to physical or cognitive impairment of the participant, and therefore was not eligible for the health literacy assessment (n = 92).

Of the 5,840 core participants in the study at wave 5, 5,330 (91.3 %) completed the health literacy assessment. Common reasons for non-completion of the health literacy assessment at wave 5 were sight problems (n = 96), health problems (n = 37), and having a study interview done by proxy (n = 214). In total, 5,256/5,840 participants had data on health literacy at both time points (90.0 %). Of these, one participant was missing data on education, two on ethnicity, and four on occupational class. Cognitive function data were missing for 257 of these participants (4.9 %). The univariate analysis of health literacy decline included all 5,256 participants, the multivariable modelling with sociodemographics included 5,252 participants, and the models including cognitive variables included 4,999 participants.

### Health Literacy

Health literacy was assessed using a four-item measure from the Adult Literacy and Life Skills Survey developed by the Organization for Economic Co-operation and Development (OECD) and Statistics Canada.^22^ Participants were required to read a fictitious medicine label similar to that found on an aspirin packet, and were asked four reading comprehension questions about the label by the interviewer (Appendix 1). Adequate health literacy was defined as scoring 4/4 correct on the measure and limited health literacy as scoring < 4/4. Health literacy decline was defined as a decrease of ≥ 1 point in score between waves.

### Cognitive Variables

Waves 2 and 5 of ELSA collection included an interviewer-administered cognitive battery, which assessed several cognitive processes essential to daily functioning that were sensitive to decline with aging and were measured in a way to prevent ceiling or floor effects.^23^ The cognitive processes assessed were: time orientation (ability to state the correct day, week, month, and year), verbal learning (of ten words presented aurally), immediate and delayed recall (of the same ten words), prospective memory (remembering to write initials on a clipboard at a certain point during the battery after being instructed to do so earlier on), verbal fluency and mental flexibility (the number of animals named in one minute), and a test of attention, visual search, and mental processing speed (the number of target letters in a grid of random alphabet letters crossed out in one minute).^23^ The former four tests were grouped to create an index of memory function, with potential scores ranging from 0 to 27, and the latter two tests were grouped to create an index of executive function, with potential scores ranging from 0 to no defined upper limit.^23^ For each index, cognitive decline was defined as a decline of > 1 point.^23^ Memory and executive function collectively will be referred to as ‘cognitive function’ and collective decline as ‘cognitive decline’ throughout this paper.

### Sociodemographic Covariates

Sociodemographic covariates obtained from the wave 2 interview were: age in years (52–54; 55–59; 60–64; 65–69; 70–74; 75–79; ≥ 80), sex (male; female), ethnicity (white; non-white), educational attainment (degree or equivalent; up to degree level; no qualification), and occupational class according to the three-category UK National Statistics Socioeconomic Classification (managerial; intermediate; routine). Age began at 52 rather than 50 years because this analysis begins 2 years into ELSA data collection. Education was included as a measure of literacy skills gained through schooling, and occupation as a measure of social standing and of literacy skills used throughout working life.

### Statistical Analysis

The prevalence of limited health literacy was calculated overall and by 5-year age group at baseline. Mean health literacy scores at each wave were calculated and graphed by 5-year age group, and compared across age groups using the Kruskal-Wallis test and within age groups using the Wilcoxon sign-rank test for matched pairs. Logistic regression models adjusted for all a priori-selected sociodemographic variables were used to estimate odds ratios (ORs) and associated 95 % confidence intervals (CIs) for the associations between age, sociodemographics, and health literacy decline. To prevent baseline adjustment bias,^24^,^25^ baseline health literacy was not adjusted for in regression modelling, as health literacy decline and score at baseline are both strongly correlated with age and likely share other common causes.

Baseline memory and baseline executive function were added to the model to determine their independent associations with health literacy decline and mediating effects on the association with age; memory decline and executive function decline were then added in a second step. A sensitivity analysis was performed, redefining cognitive decline variables according to increasingly conservative definitions of decline: decreases of > 2 and > 5 points on each index. A post-hoc analysis of chronic disease diagnoses that may affect cognition was run to assess their potential additional contribution to explaining the association between age and health literacy. Chronic diseases were diabetes, heart disease (angina, heart attack, abnormal heart rhythm or congestive heart failure), chronic lung disease (chronic bronchitis or emphysema), and depressive symptoms.

The impact of missing data was investigated by running multiple imputations of missing values for health literacy and cognitive function (see full Methods in Appendix 2). All analyses were conducted using StataSE 13.1 (StataCorp, College Station, TX).

## RESULTS

**Figure 1.**
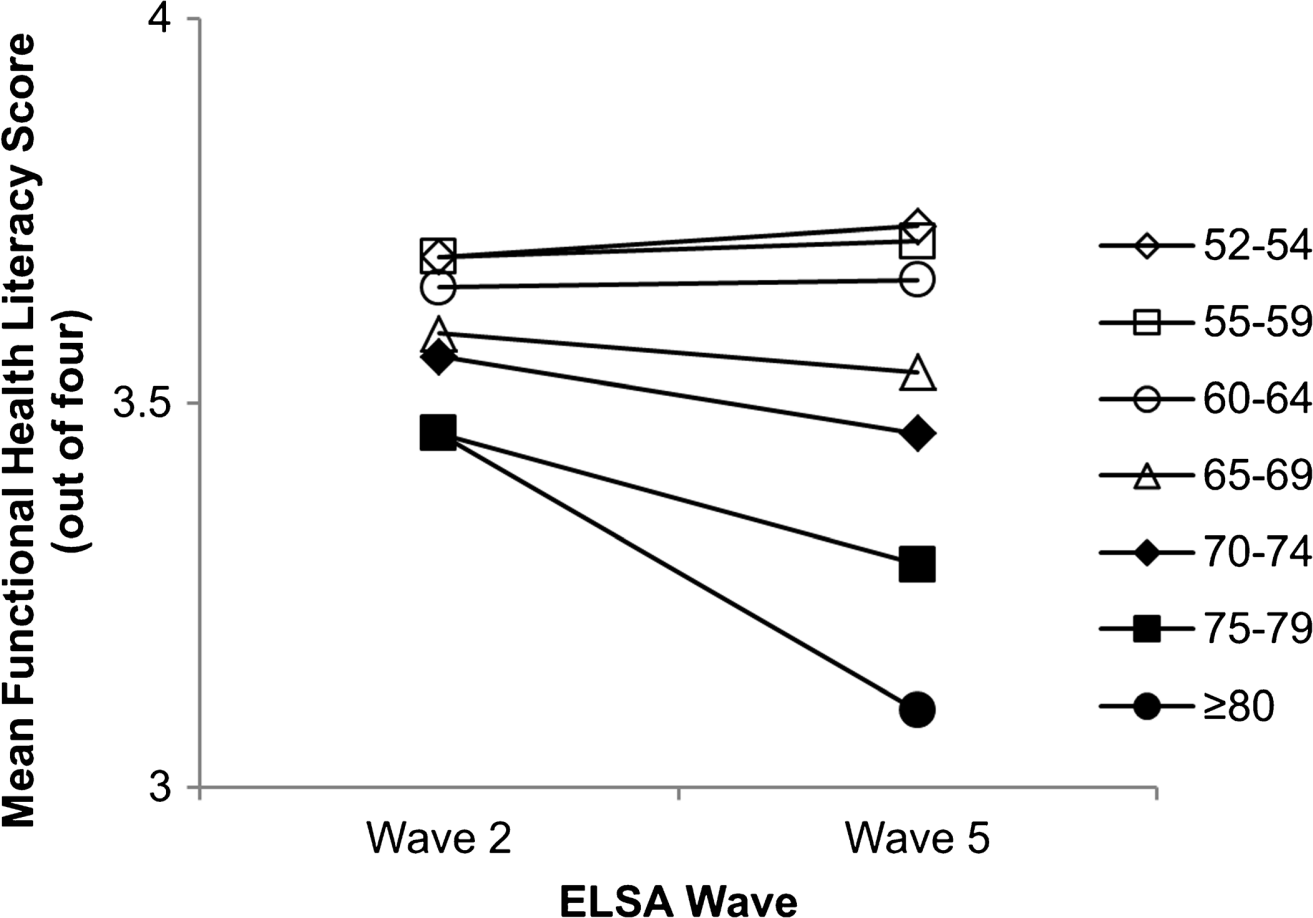
Mean health literacy scores between ELSA waves 2 (2004–2005) and 5 (2010–2011) by 5-year age group.

At baseline, 1,455/5,256 (27.7 %) participants had limited health literacy (Table 1). When followed forward to wave 5, 3,260/5,256 participants (62.0 %) had no change in their health literacy score, while 964/5,256 (18.3 %) improved by ≥ 1 point and 1,032/5,256 (19.6 %) declined by ≥ 1 point. Chi-squared tests showed that improvement in score was non-differential by age (*p* = 0.53), while decline was more frequent in older age groups (*p* < 0.001). The proportion that declined increased linearly with age from 14.8 % (78/526) of those aged 52–54 years (102/267) to 38.2 % of those aged ≥ 80 years (*p* < 0.0001). As shown in Fig. 1, mean health literacy scores declined over the study follow-up for age groups from 65–69 years and older; this decline was statistically significant for the 75–79 (*p* = 0.008) and ≥ 80 (*p* < 0.001) groups.

**Table 1.** Baseline Characteristics of Study Participants, the English Longitudinal Study of Ageing, 2004–2005 (n = 5,256)

	N (%)
Age	
52–54	526 (10 %)
55–59	1,346 (26 %)
60–64	995 (19 %)
65–69	952 (18 %)
70–74	692 (13 %)
75–79	478 (9 %)
≥ 80	267 (5 %)
Health literacy	
Limited	1,455 (28 %)
Sex	
Female	2,960 (56 %)
Ethnicity	
Non-white	85 (2 %)
Occupational class	
Managerial	1,797 (34 %)
Intermediate	1,364 (26 %)
Routine	2,040 (39 %)
Other	53 (1 %)
Educational attainment	
Degree or equivalent	1,268 (24 %)
Up to degree level	2,349 (45 %)
No qualification	1,635 (31 %)
Memory index (/27)	
Mean (SD)	16.57 (3.74)
Median	17
Range	4–27
Executive function index	
Mean (SD)	13.66 (3.10)
Median	14
Range	4–23

In the multivariable logistic regression model adjusted for sex, ethnicity, occupation, and education, the OR for health literacy decline among those aged 65–69 years vs. 52–54 years was 1.34 (95 % CI: 1.00–1.79; Table 2). ORs increased in a linear fashion across age groups (*p*_trend_ < 0.001) up to 3.21 (95 % CI: 2.26–4.57) for the ≥ 80 vs. the 52–54 age group. Independent of baseline age, the sociodemographic risk factors for health literacy decline were: being male (OR = 1.20; 95 % CI: 1.04–1.38), of a non-white ethnicity (OR = 2.42; 95 % CI: 1.51–3.89), being in an occupational class lower than professional/managerial (OR = 1.35; 95 % CI: 1.11–1.65 for intermediate; OR = 1.67; 95 % CI: 1.39–2.01 for routine; OR = 1.90; 95 % CI: 1.02–3.53 for ‘other’), and having no educational qualifications (OR = 1.58; 1.29–1.95).

**Table 2.** Multivariable-Adjusted Associations Between Age, Sociodemographic Factors, Cognition, and Health Literacy Decline, the English Longitudinal Study of Ageing, England, 2004–2011 (n = 5256)

	Odds ratios for health literacy decline		
	OR^*^ (95 % CI)	OR^†^ (95 % CI)	OR^‡^ (95 % CI)
Age			
52–54	1.00 (Reference)	1.00 (Reference)	1.00 (Reference)
55–59	0.92 (0.69–1.23)	0.95 (0.70–1.28)	0.93 (0.68–1.26)
60–64	1.07 (0.80–1.44)	1.04 (0.76–1.41)	0.98 (0.72–1.34)
65–69	1.34 (1.00–1.79)	1.21 (0.90–1.65)	1.08 (0.79–1.48)
70–75	1.53 (1.13–2.07)	1.26 (0.91–1.74)	1.07 (0.77–1.48)
75–79	1.94 (1.41–2.67)	1.43 (1.01–2.03)	1.09 (0.76–1.55)
≥ 80	3.21 (2.26–4.57)	2.26 (1.54–3.32)	1.65 (1.11–2.45)
Sex			
Male	1.20 (1.04–1.38)	1.10 (0.94–1.27)	1.05 (0.90–1.22)
Ethnicity			
Non-white	2.42 (1.51–3.89)	1.81 (1.10–2.99)	1.67 (1.01–2.77)
Occupational class			
Managerial	1.00 (Reference)	1.00 (Reference)	1.00 (Reference)
Intermediate	1.35 (1.11–1.65)	1.26 (1.02–1.55)	1.24 (1.01–1.53)
Routine	1.67 (1.39–2.01)	1.50 (1.23–1.82)	1.43 (1.18–1.74)
Other	1.90 (1.02–3.53)	1.60 (0.83–3.06)	1.44 (0.75–2.77)
Educational attainment			
Degree or equivalent	1.00 (Reference)	1.00 (Reference)	1.00 (Reference)
Up to degree level	1.04 (0.86–1.26)	1.06 (0.87–1.30)	1.07 (0.88–1.31)
No qualification	1.58 (1.29–1.95)	1.33 (1.07–1.66)	1.30 (1.04–1.62)
Baseline memory			
Per 1-point score increase	–	0.96 (0.93–0.98)	0.94 (0.91–0.96)
Baseline executive function			
Per 1-point score increase	–	0.93 (0.91–0.96)	0.92 (0.89–0.94)
Memory decline			
Yes	–	–	1.59 (1.35–1.87)
Executive function decline			
Yes	–	–	1.56 (1.32–1.85)

^*^Adjusted for covariates (age, sex, ethnicity, occupational class, educational attainment)

^†^Adjusted for covariates, and baseline memory & executive function; n = 4999

^‡^Adjusted for covariates, baseline memory & executive function, and memory & executive function decline; n = 4999

Mean baseline memory and executive function decreased with age (*p* < 0.0001). Higher baseline cognitive function was protective against health literacy decline regardless of age, where every 1-point increase in memory score was associated with an OR of 0.94 (95 % CI: 0.91–0.96) and every 1-point increase in executive function score was associated with an OR of 0.92 (95 % CI: 0.89–0.94) for health literacy decline (Table 2). The likelihood of cognitive decline over the follow-up period increased with age: 30.9 % (162/525) of those aged 52–54 experienced memory decline, compared with 55.2 % (144/261) of those aged ≥ 80; the corresponding values for executive function decline were 25.6 % (128/500) and 45.3 % (111/245) (*p* < 0.001 for both). As shown in Table 2, memory and executive function decline were associated with health literacy decline independent of age (ORs = 1.59; 95 % CI: 1.35–1.87 and 1.56; 95 % CI: 1.32–1.85). Baseline cognitive function and cognitive decline over the follow-up period explained most of the association between health literacy decline and age. The associations between other sociodemographic variables and health literacy decline persisted regardless of adjustment for cognition (Table 2).

When memory decline was defined as declines of > 2 and > 5 points, 1,386/4,999 (27.7 %) and 485/4,999 (9.7 %) participants were defined as experiencing memory decline. The corresponding values for these re-definitions of executive function decline were 988/4,999 (19.8 %) and 138/4,999 (2.8 %). When each of these increasingly conservative definitions was used to create the cognitive decline variables, results were unaltered from the original analysis. Having a chronic disease diagnosis or depressive symptoms was not associated with health literacy decline when added to the final model. Finally, results from the multiple imputation analysis were mostly similar to the complete-case analysis (Appendix 2).

## DISCUSSION

Nearly one-third of English adults aged 52 years and over had health literacy limitations in this large longitudinal study. Over the 6-year follow-up period, one-fifth of the sample declined in health literacy skills. Age differences in the likelihood and rate of health literacy decline were pronounced, with adults over age 80 having over three times greater odds of experiencing health literacy decline than those in their early 50s. Striking social inequalities in health literacy decline were evident, where men, ethnic minorities, those with no educational qualifications, and those with a lower occupational class were vulnerable to loss of the literacy skills required to manage health during aging. Cognition appears to be a key risk factor explaining health literacy decline. Even subtle, one-point differences in cognitive function affected the likelihood of health literacy decline, and experiencing cognitive decline of any magnitude was strongly associated with health literacy decline.

This study is the first and the largest to our knowledge to track health literacy skills over time, particularly among an aging sample. Our finding that cognitive function mostly explained the relationship between older age and health literacy decline was expected, based on cross-sectional evidence showing that the constructs of cognition and health literacy overlap to a large degree.^12^,^13^,^18^,^26^ Contrary to our findings, the association between age and health literacy was independent of cognitive impairment according to the Mini Mental Status Examination (MMSE) score in previous research,^14^,^16^,^17^ although the MMSE does not detect subtle individual differences in cognitive function. An important aspect of our study is that not everyone who experienced cognitive decline also experienced health literacy decline. The degree to which typical cognitive aging versus aging-related cognitive impairments of varying severities affect health literacy skills remains to be elucidated. Our study suggests that non-pathological cognitive decline negatively affects health literacy during aging. Further longitudinal studies that address the fluidity of literacy and cognition during aging are needed for consideration alongside ours.

We observed a degree of health literacy decline among adults aged ≥ 80 years not explained by cognition, which may be because we could not account for all aspects of cognitive function. For example, inductive reasoning was not measured, but is correlated with both age and health literacy.^12^,^27^ Visual and auditory functioning also play roles in one’s ability to take in and learn from new information. We did not account for these factors, although participants unable to take the test due to sensory limitations were excluded. We also had no measures of the component processes involved with active learning, including knowledge integration and text inference, which predict reading comprehension skills among older adults.^28^ However, the aspects of short-term memory and processing speed that we measured are related to these abilities. It may also be that other factors besides cognitive function influence potential generational differences in likelihood of literacy skill loss, such as lifetime educational experiences.

Although validation data for the individual health literacy measure we used were not available, the measure was taken from a validated international adult literacy survey.^22^ The measure does not capture prose literacy, information navigation, or numeracy, although it is a measure of document literacy that has good face validity. The ability to read and understand a medicine label is crucial to several health outcomes, and has been associated with risk of all-cause mortality among older adults.^5^ The scale had narrow range and a ceiling effect, where over two-thirds of our study sample scored 4/4 on the scale at both time points; this is a common problem in health literacy measures.^29^,^30^ Consequently, few participants declined by > 1 point (only 316/5,256; 6 %), preventing us from examining decline of varying magnitudes and from varying starting points. We could not examine non-linear change or change longer than our 6-year follow-up period. However, as a longitudinal analysis conducted with little prior knowledge on health literacy during aging, this study provides valuable evidence for future research hypotheses.

Another important limitation of this study is attrition bias. The prevalence of limited health literacy at baseline was 42 % among those who dropped out of the study, but was only 28 % among those who remained in the study between waves. Study attrition also increased with age, from approximately 26 % among those aged 52–54 years to 71 % among those aged ≥ 80 years. Our results may therefore underestimate the true prevalence of limited health literacy among the older English population, particularly in the most elderly age group. Ethnic minorities, participants with no educational qualifications, and those with routine occupations were more also likely to drop out of the study, and were more likely to have limited health literacy at baseline. Therefore, we may have underestimated the magnitude of associations between these sociodemographic variables and health literacy decline. Reassuringly, missing data do not seem to introduce notable bias into our results, as results from the multiple imputation analysis were similar to those from the complete-case analysis.

Future work should investigate more comprehensive aspects of cognition including reasoning and sensory functioning to elucidate the role of cognition in health literacy decline. Longitudinal data should be collected at multiple time points to examine non-linear trajectories of health literacy change over time and for longer follow-up periods than 6 years. Potentially modifiable behavioral and health-related influences on health literacy are unknown. For example, internet use and engagement in regular reading may help adults to maintain health literacy during aging through directly stimulating cognitive and literacy skills. Diagnoses of health conditions, physical functioning, and experiences with the health care system may affect health literacy in multiple complex ways. Future research should investigate these and other potential influences on health literacy decline; if certain practices can help maintain health literacy skills regardless of cognitive aging, this evidence will inform the development of interventions to improve literacy skills in health settings.

## CONCLUSIONS

The literacy skills required to manage health appear to undergo aging-related decline among older English adults beginning around age 65. Rate of decline increases with age, with adults aged ≥ 80 years being vulnerable to rapid health literacy decline. Health literacy among older adults is marked by social inequalities, whereby men and adults from deprived social groups are the most vulnerable to skill loss during aging. Cognitive function and even slight cognitive decline during aging appear to affect the likelihood of health literacy decline. Finally, given that literacy skills are commonly lost during aging, a time when adults often need health information and services, the current population-wide burden of low health literacy may be substantial. Innovative interventions to help reduce and prevent literacy barriers to good health during aging are needed, along with individual support from practitioners in daily practice.

## Electronic supplementary material

Below is the link to the electronic supplementary material.

ESM 1 (DOCX 84 kb)
