# Supplementary material for: Cognitive Function and Health Literacy Decline in a Cohort of Aging English Adults
Source: J Gen Intern Med. 2015 Feb 14;30(7):958–64. doi: 10.1007/s11606-015-3206-9 (PMC4471026; doi:10.1007/s11606-015-3206-9)
Supplement: Supplementary file 1 — (DOCX 84 kb) [file 11606_2015_3206_MOESM1_ESM.docx]

**APPENDICES**

**Appendix 1: Health literacy assessment**

INSTRUCTIONS READ OUT BY THE INTERVIEWER

The final task in this section is about comprehension. This is a made-up medicine label and does not refer to a real medicine. It is often difficult to read and understand instructions on medicine labels. In a moment, I will ask you to read the card quietly to yourself. I will then ask you some questions about what it says. You do not have to memorise the card, as you will be able to look at it while answering the questions.

1. What is the maximum number of days you may take this medicine?

*(Correct answer 7. If responds with 'one week', interviewer may probe for number of days. Other answers incorrect.)*

1. List three situations for which you should consult a doctor.

*(Respondent should mention at least three of the following: (Before giving medication to children with) chicken pox, (Before giving medication to children with) influenza, Reyes syndrome, (During) lactation, (During) pregnancy, If symptoms persist, (Accidental) overdose. Incorrect answer: any other response.)*

1. List one condition for which you might take the Medco tablet.

*(Correct if answered one of: Headaches, Muscle pains, Rheumatic pains, Toothache, Earache, Common cold. Other answers incorrect.)*

1. List one condition for which you should not take the Medco tablet.

*(Correct if respondent mentions at least one of the following as conditions for which you should not take the tablet: Gastritis, Peptic ulcer, Serious liver illness, Bronchial asthma. Incorrect answer: any other response.)*

Scoring: 1 point per complete correct response.

**Appendix 2: Multiple imputation for missing data**

In the ‘core’ ELSA sample, 5840/8780 (67%) participants were retained between waves 2 and 5 and were eligible for the present analysis. Of these 5840 participants, 186 (3.2%) were missing health literacy data at wave 2, 510 (8.7%) were missing health literacy data at wave 5, 51 (0.9%) were missing memory index data at wave 2, 168 (2.9%) were missing executive function index data at wave 2, 401 (6.9%) were missing memory index data at wave 5, and 645 (11.0%) were missing executive function index data at wave 5. Little’s MCAR test showed that health literacy data were not missing completely at random (χ^2^(4)=262.57, *P*<0.0001), and neither were cognitive function data missing at random (χ^2^(37)=530.21, *P*<0.0001). Data were not missing in a monotone fashion. Missing values were imputed using a multivariate normal regression (an iterative Markov Chain Monte Carlo method) to create 20 imputed datasets. Absolute health literacy score at waves 2 and 5, and absolute scores for the memory and executive function indices at waves 2 and 5 were imputed from the estimation model, which included age at wave 2, sex, ethnicity, occupational class, educational attainment, and self-rated sight, as sight problems were a common cause of non-completion of the health literacy assessment. The estimation model excluded 29 out of 5840 observations because all imputation variables were missing for these individuals. Health literacy decline and cognitive decline (memory and executive function) were specified as ‘passive’ variables in the multiple imputation dataset, meaning that their values are wholly dependent on the imputed variables; the Stata command ‘mi passive: replace’ was used to calculate their values from the imputed dataset. The final logistic regression model using the imputed data had 5833 participants due to missing covariate data. The Supplementary Table below shows the results.

| Supplementary Table. Multivariable logistic regression results with multiple imputation for missing values of health literacy and cognition (n=5833) | | | |
| --- | --- | --- | --- |
|  | Odds ratios for health literacy decline | | |
|  | OR^*^ 95% CI | OR^†^ 95% CI | OR^‡^ 95% CI |
| Age |  |  |  |
| 52-54 | 1.00 | 1.00 | 1.00 |
| 55-59 | 0.99 (0.75-1.31) | 0.94 (0.71-1.25) | 0.92 (0.69-1.22) |
| 60-64 | 1.13 (0.84-1.51) | 0.99 (0.74-1.33) | 0.94 (0.70-1.27) |
| 65-69 | 1.36 (1.03-1.80) | 1.14 (0.86-1.52) | 1.03 (0.77-1.37) |
| 70-75 | 1.65 (1.24-2.22) | 1.27 (0.94-1.71) | 1.08 (0.80-1.47) |
| 75-79 | 2.20 (1.62-2.98) | 1.56 (1.13-2.14) | 1.20 (0.87-1.66) |
| ≥80 | 4.25 (3.08-5.87) | 2.57 (1.83-3.61) | 1.88 (1.33-2.66) |
| Sex |  |  |  |
| Male | 1.20 (1.05-1.39) | 1.10 (0.95-1.27) | 1.06 (0.92-1.23) |
| Ethnicity |  |  |  |
| Non-white | 2.09 (1.31-3.32) | 1.47 (0.91-2.37) | 1.36 (0.84-2.19) |
| Occupational class |  |  |  |
| Managerial | 1.00 | 1.00 | 1.00 |
| Intermediate | 1.28 (1.06-1.54) | 1.19 (0.98-1.44) | 1.18 (0.97-1.43) |
| Routine | 1.58 (1.32-1.88) | 1.37 (1.14-1.64) | 1.31 (1.09-1.57) |
| Other | 1.84 (1.04-3.23) | 1.58 (0.89-2.83) | 1.47 (0.83-2.64) |
| Educational attainment |  |  |  |
| Degree or equivalent | 1.00 | 1.00 | 1.00 |
| Up to degree level | 0.98 (0.81-1.18) | 0.96 (0.79-1.16) | 0.96 (0.80-1.17) |
| No qualification | 1.51 (1.25-1.83) | 1.25 (1.03-1.53) | 1.21 (0.99-1.48) |
| Baseline memory |  |  |  |
| Per 1-point score increase |  | 0.95 (0.93-0.97) | 0.93 (0.91-0.95) |
| Baseline executive function |  |  |  |
| Per 1-point score increase |  | 0.93 (0.91-0.95) | 0.92 (0.89-0.94) |
| Memory decline |  |  |  |
| Yes |  |  | 1.58 (1.35-1.84) |
| Executive function decline |  |  |  |
| Yes |  |  | 1.50 (1.27-1.77) |

^*^ Adjusted for covariates (age, sex, ethnicity, occupational class, educational attainment)

^†^ Adjusted for covariates and baseline memory & executive function

^‡^ Adjusted for covariates, baseline memory & executive function, and memory & executive function decline
